# SNP array-based analyses of unbalanced embryos as a reference to distinguish between balanced translocation carrier and normal blastocysts

**DOI:** 10.1007/s10815-016-0734-0

**Published:** 2016-05-30

**Authors:** Nathan R. Treff, Katherine Thompson, Michael Rafizadeh, Michael Chow, Liza Morrison, Xin Tao, Heather Garnsey, Christine V. Reda, Talia L. Metzgar, Shelby Neal, Chaim Jalas, Richard T. Scott, Eric J. Forman

**Affiliations:** 1 Reproductive Medicine Associates of New Jersey, Basking Ridge, NJ 07920 USA; 2 Rutgers-Robert Wood Johnson Medical School, New Brunswick, NJ 08901 USA; 3 Foundation for Embryonic Competence, Basking Ridge, NJ 07920 USA

**Keywords:** Reciprocal translocation, Microarray, Preimplantation genetic testing

## Abstract

**Purpose:**

The purpose of the study is to validate a method that provides the opportunity to distinguish a balanced translocation carrier embryo from a truly normal embryo in parallel with comprehensive chromosome screening (CCS).

**Methods:**

A series of translocation carrier couples that underwent IVF with single nucleotide polymorphism (SNP) array-based CCS on 148 embryos were included. Predictions of balanced or normal status of each embryo were made based upon embryonic SNP genotypes. In one case, microdeletion status was used to designate whether embryos were balanced or normal. In 10 additional cases, conventional karyotyping was performed on newborns in order to establish the true genetic status (balanced or normal) of the original transferred embryo. Finally, implantation potential of balanced or normal embryos was compared.

**Results:**

Phasing SNPs using unbalanced embryos allowed accurate prediction of whether transferred embryos were balanced translocation carriers or truly normal in all cases completed to date (100 % concordance with conventional karyotyping of newborns). No difference in implantation potential of balanced or normal embryos was observed.

**Conclusions:**

This study demonstrates the validity of a CCS method capable of distinguishing normal from balanced translocation carrier embryos. The only prerequisite is the availability of parental DNA and an unbalanced IVF embryo, making the method applicable to the majority of carrier couples. In addition, the SNP array platform allows simultaneous CCS for aneuploidy with the same platform and from the same biopsy. Future work will involve prospective predictions to select normal embryos with subsequent karyotyping of the resulting newborns.

## Introduction

Very few causes of infertility or recurrent pregnancy loss can be explained by a clear genetic origin. Chromosomal rearrangements represent one such cause with carrier patients experiencing an increased rate of failure to achieve a pregnancy and likelihood of having a miscarriage due to the unbalanced transmission of derivative chromosomes to gametes and offspring. One option available to these individuals is provided by in vitro fertilization (IVF) with preimplantation genetic testing (PGT). Embryos that inherit an unbalanced karyotype can be readily identified through a variety of testing methods including fluorescence in situ hybridization, array comparative genomic hybridization, and single nucleotide polymorphism microarrays.

The array-based methods of testing provide the added benefit of screening for whole chromosome aneuploidy of all 24 chromosomes (22 autosomes, X and Y) in parallel with the identification of unbalanced inheritance of chromosomes from the translocation [1–5]. Several randomized controlled trials have already demonstrated that comprehensive chromosome screening (CCS) for aneuploidy significantly improves clinical outcomes [6–9] such that the same improvement can be expected when applied to patients who carry a balanced translocation. Previous studies investigating the application of array-based CCS methods in patients with translocations indicate similar maternal age-related increase in aneuploidy of chromosomes unrelated to the translocation chromosomes. In addition, these studies have also helped determine the resolution of detection of segmental aneuploidies associated with the translocation [10]. Unbalanced embryos and embryos with aneuploidy for other chromosomes can be identified and excluded from selection for embryo transfer in order increase success rates and avoid chromosomally related pregnancy loss.

One of the remaining challenges to PGT with CCS in embryos derived from translocation carrier patients is the inability to distinguish an embryo that is a carrier of the balanced translocation from one that is truly normal [11]. Current methods are therefore unable to prevent inheritance of the balanced translocation in the children born following IVF with PGT and CCS. Many patients would prefer to avoid transmitting their translocation and its associated risk of infertility and miscarriage to their offspring. This is particularly true if no additional cost were to be incurred in the process of making a distinction between the two types of embryos.

Despite this, it has remained challenging to distinguish between a balanced translocation carrier embryo and one that is truly normal while also providing CCS for aneuploidy in parallel. This is in part due to the indistinguishable nature of the results obtained from either type of embryo. Both a balanced translocation carrier embryo and one that is truly normal display the same copy number neutrality. That is, when the copy number analysis is obtained from array-based methods commonly used for testing, both types of samples give the exact same profile, a copy number of 2.

A previously validated quantitative SNP array-based CCS method [12] can reliably provide both copy number and genotyping data and has demonstrated the ability to resolve segmental aneuploidies as small as 2.36 Mb for translocation patients [5, 13]. This unique ability may enable distinguishing balanced from normal embryos while also providing CCS. In a manner very similar to establishing linked markers for single gene disorder PGD, SNPs which are near the translocation breakpoints and also heterozygous (two different alleles) in the carrier parent, and homozygous in the partner, could be used to track which chromosomes are inherited in the copy number neutral (normal or balanced) embryos by determining which of the two alleles are present. Unbalanced embryos could also be used to determine which of the two alleles are linked to the derivative or to the normal chromosomes. The ability to predict actual status from embryo biopsies could then be validated by a blinded comparison to conventional karyotypes performed on children born after IVF and PGT with this SNP array methodology.

## Materials and methods

### Strategy

**Fig. 1:**
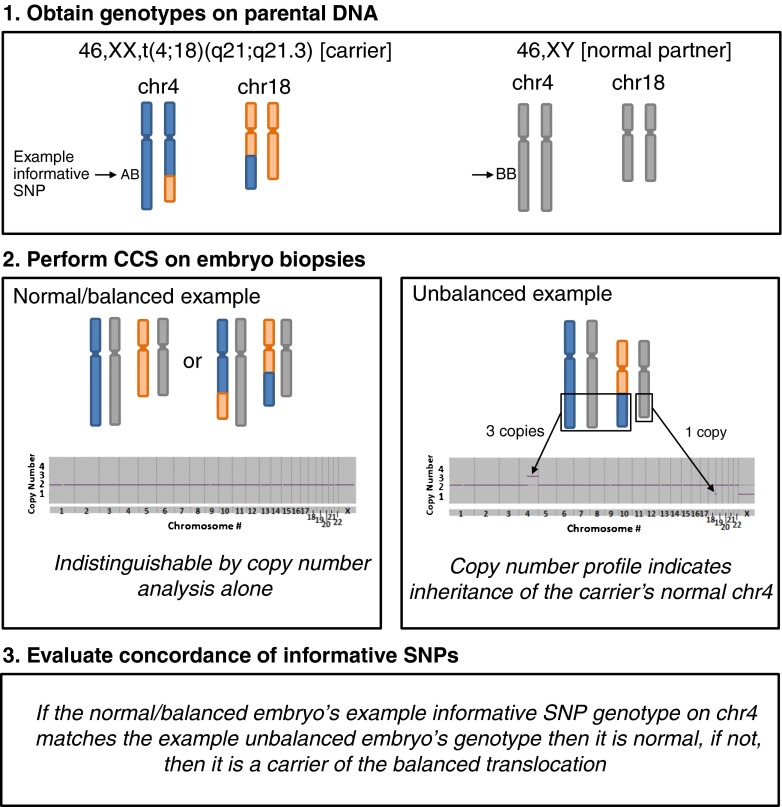
Workflow diagram for using SNP genotyping information from parental DNA and an unbalanced embryo to determine the carrier status of copy number neutral embryos

This study was organized into three phases. Phase 1 involved evaluating whether the method (Fig. 1) of predicting balanced or normal embryos was consistent with the presence of a microdeletion in a previously published case. In this case, unlike most translocation cases, a microdeletion near the breakpoint of one of the translocations was present in the patient and known to cause Alagille syndrome. If present in an embryo, the microdeletion would indicate that it was also a carrier of the translocation. Phase 2 involved using the same method to make predictions in a series of more conventional translocation patients who had already undergone IVF with PGT and embryo transfer of a balanced/normal embryo. Newborn karyotypes were obtained to determine the true status of the embryos (balanced or normal) in order to evaluate the level of concordance of the SNP genotyping methodology of prediction from the original embryo biopsy. Phase 3 involved using the validated prediction method to determine whether balanced carrier embryos implant less often than truly normal embryos.

### SNP array analyses

Default settings of GTYPE were used to obtain genotypes for parental DNA using the Affymetrix NspI GeneChip as recommended by the supplier. “Informative” SNPs were defined as those that were heterozygous in the carrier patient and homozygous in the partner within 5 Mb of the breakpoints for each chromosome involved in the translocation. This distance was chosen in order to avoid misinterpretation from possible recombination events that might occur during meiosis in the carrier patient’s gametes.

Embryo trophectoderm biopsies were processed with whole genome amplification (WGA) and SNP array analysis as previously described [12]. The same informative SNPs were compared between embryos identified as copy number normal (balanced/normal) to embryos identified as unbalanced. An example of this process is illustrated in Fig. 1. Genotyping was performed with settings of GTYPE as previously described. Briefly, settings were adjusted in order to obtain more accurate genotype predictions but with fewer successful genotypes per SNP tested. Unbalanced embryos that possessed one normal chromosome and one derivative chromosome were identified using copy number analysis as previously described. When a chromosome from a balanced/normal embryo was found to be more similar to an unbalanced embryo derivative chromosome, the embryo was predicted to be a carrier of the balanced translocation. When a chromosome from a balanced/normal embryo was found to be more similar to an unbalanced embryo normal chromosome, the embryo was predicted to be normal. In each case, the balanced/normal embryo provided two measurements of similarity, one for each of the two chromosomes involved in the translocation. In addition, when multiple unbalanced embryos were available for comparison, all such comparisons were performed.

### Newborn karyotyping

Children born following the conventional use of SNP array-based testing for CCS and unbalanced translocations had conventional G-banding karyotypes performed from peripheral blood. Reports were obtained and then compared to the blinded predictions made from SNP array analysis of the transferred embryos as described above.

## Results

### Phase 1

In order to establish the potential validity of the proposed methodology, a specific case with previously published data was used prior to recruiting new patients to participate. Since this particular case had a unique microdeletion associated with the balanced translocation, newborn karyotyping was not needed in order to have a way to determine the actual status of each embryo (balanced or normal). That is, in each embryo, the presence or absence of the microdeletion could be used to determine whether the embryo was a carrier of the balanced translocation or normal, respectively. In this case, a total of 12 embryos were originally found to be balanced or normal using the conventional copy number analysis of the SNP array data. Informative SNPs within 5 Mb of the breakpoints on each of the two chromosomes involved in the translocation were identified by analysis of parental DNA. A comparison of the genotypes at these informative SNP positions was performed for each balanced/normal embryo against all possible unbalanced embryos. In 10 cases, the similarities indicated that the embryos were normal, and in 2 cases, the embryos were consistent with a balanced translocation carrier status. The microdeletion status of each of the balanced/normal embryos was then unblinded and compared to the SNP genotyping-based predictions. All 12 cases were concordant. Therefore, the first phase of this study indicated 100 % accuracy of using unbalanced embryos and SNP genotyping analyses to predict whether balanced/normal embryos were actually balanced carriers or truly normal.

### Phase 2

Based upon these findings, 10 patients who had already undergone IVF with SNP array-based testing of their embryos for CCS and unbalanced translocations were successfully recruited. Informative SNPs within 5 Mb of the breakpoints in embryos which were selected for transfer and led to delivery were then evaluated against unbalanced embryos from the same patient. In 2 cases, the embryos were predicted to be carriers of a balanced translocation, and in 8 cases as truly normal. Karyotypes were obtained from newborns in each of the 10 cases and confirmed the embryo SNP genotyping-based prediction of the genetic status of the embryo in all cases (100 % concordance).

### Phase 3

Having validated the ability to distinguish balanced from normal embryos using SNP genotyping from an embryo biopsy, we applied the technique to evaluate the implantation potential of both types of embryos. Out of 126 embryos evaluated, 62 (49 %) were predicted to be normal and 64 (51 %) were predicted as balanced carriers of a translocation. Forty-two (68 %) of the 62 normal embryos and 38 (59 %) of the 64 carrier embryos successfully implanted, which was not significantly different (chi-square *p* value = 0.33). Maternal age and number of oocytes retrieved were also not significantly different amongst the two groups of embryos.

## Discussion

IVF with PGT has provided a powerful option for couples who carry a balanced translocation and has allowed them to avoid experiencing miscarriages due to unbalanced embryos. Without the benefit of a conventional karyotype to see the structure of the rearrangement, it has been impossible to make a prediction of whether the embryo is balanced vs. normal while also providing CCS data. Even arguably more powerful technologies like next-generation sequencing would not be able to solve this problem as they would predict a copy number of 2 in either case for the affected chromosomes. Therefore, despite having a successful IVF cycle and delivery, many of these couples will be passing on the translocation to their children who may also be subjected to infertility, recurrent pregnancy loss, and, perhaps, have to use assisted reproductive technologies to conceive. The couples who participated in this IRB-approved study all expressed a desire to select against the translocation and remove it from their reproductive lineage, sparing their future children of the reproductive challenges they faced. To date, this option has not been reliably available.

Using a SNP array platform for CCS allows reliable differentiation between balanced and normal embryos without significant additional testing or expense. New probes do not need to be developed and treatment need not be delayed. Rather SNP genotyping data from the parents can be used in combination with the data from unbalanced embryos to provide a prediction prior to frozen embryo transfer. Ongoing research will continue to further validate this approach. However, the blinded prediction being correct in 10 out of 10 cases, which would be 1024 to 1 odds of happening by chance, is very reassuring.

Interestingly, approximately half of the transferred embryos were normal vs. balanced indicating that morphology-based selection from amongst the transferrable embryos will not reliably select against the balanced translocation. There was a small, but not significant, increase in implantation rates amongst normal embryos; ongoing research will continue to evaluate whether there is a significant difference. This method relies on the availability of an unbalanced embryo being present. In those cases without an unbalanced embryo, the couple could choose to transfer one by morphology and the karyotype of the newborn could be used to make predictions prior to subsequent transfers.

This study provides validation for a method capable of providing both CCS and the ability to identify embryos that are carriers of balanced translocations. While much of the field of PGS has moved to non-genotyping-based methods of CCS (qPCR and array CGH), this study demonstrates a unique application for SNP array-based testing in embryos from patients carrying a balanced translocation.
